# Integrated care pathways for Black persons with traumatic brain injury: a protocol for a critical transdisciplinary scoping review

**DOI:** 10.1186/s13643-020-01323-8

**Published:** 2020-06-01

**Authors:** Samira Omar, LLana James, Angela Colantonio, Stephanie A. Nixon

**Affiliations:** 1 Rehabilitation Sciences Institute, Faculty of Medicine, University of Toronto, 500 University Avenue, Toronto, ON M5G 1V7 Canada; 2 Department of Occupational Science and Occupational Therapy and Rehabilitation Sciences Institute, University of Toronto, 160-500 University Ave, Toronto, ON M5G 1V7 Canada; 3 Department of Physical Therapy and Rehabilitation Sciences Institute, University of Toronto, 160-500 University Ave, Toronto, ON M5G 1V7 Canada

**Keywords:** Traumatic brain injury, Integrated care pathways, Critical transdisciplinary, Black, Blackness, Racism, Racialization, Critical transdisciplinary scoping review

## Abstract

**Background:**

Current understandings of the etiology of traumatic brain injury (TBI) and the trajectory of care significantly lack consideration for the inclusion of Black populations. The global prevalence of TBI is increasing, particularly in North America and Europe where approximately 65 million people are affected every year. Although community integration is an ultimate goal of rehabilitation post injury, persons with TBI, particularly Black populations continually face challenges with regards to unmet needs along the continuum of care including meaningful participation and vocation, resulting in occupational deprivation. While integrated care is seen as an appealing approach to service delivery, little is known about what this means for Black people with TBI. This protocol produces the first critical transdisciplinary (CTD) scoping review mapping the extent, range, and nature of integrated care pathways for Black people experiencing TBI.

**Methods:**

CTD provides an analytical tool with a health equity lens that will be applied as both a methodology and theory for undertaking this review. Under the methodological guidance of Arksey and O’Malley, CTD will be used to map the literature and better understand the elements of integrated care pathways for Black people experiencing TBI. To identify the published literature, several databases will be searched including MEDLINE, EMBASE, Cochrane Central Register of Controlled Trials, CINAHL, PsycINFO, and Sociological Abstracts.

**Discussion:**

The application of CTD compels health-care providers, administrators, clinician-scientists, rehabilitation specialists, and scholars in the field of TBI and integrated care to re-examine hidden assumptions about racism, racialization, and Blackness that are often embedded in current visions of health for all. The health equity lens of CTD asks about who is accounted for in the research and clinical literature and who is absented. It is anticipated that applying the health equity lens of CTD will provide a critical examination of the literature and illuminate significant implications for integrated care for Black persons experiencing TBI.

**Systematic review registration:**

Not applicable.

## Background

Wilson [1] describes traumatic brain injury (TBI) as an underappreciated public health concern. Current understandings of the injury prevalence, etiology of disease, care, recovery, and community integration lack consideration of the role of structural or systemic forces that is described by Raphael [2, 3]. A key shortcoming is interpretation of the impact of racialization, racism, and its intersections, which have not been addressed despite the influence of colonial histories in North America and globally [4, 5]. This protocol describes the rationale and methodology for a scoping study that maps the extent, nature, and range of the literature on integrated care pathways for Black people experiencing TBI.

The U.S. Center for Disease Control and Prevention (CDC) defines TBI as a disruption or change in brain function “that can be caused by a bump, blow, or jolt to the head” [6]. TBI can range from a mild concussion to a severe head injury. Mild TBI accounts for an estimated total of 59.9 million cases each year, and severe TBI affects approximately 5.48 million people yearly [7]. Over the last three decades, the epidemiology and clinical characteristics of persons with TBI have significantly changed as mortality rates have decreased by approximately 50% [8], due to advances in health-care technology. However, as a chronic disease, TBI affects 69.1 million people every year [7] and is more common than breast cancer, HIV/AIDS, spinal cord injury, and multiple sclerosis combined [9]. Globally, the incidence of TBI recorded in clinical settings is the highest in North America, with 1299 cases per 100,000 people [7].

The consequences of TBI may involve an assemblage of changes with physical, psychosocial, cognitive, behavioral, and emotional states [10, 11]. These changes have been shown to have a notable impact on occupational performance including vocation, leisure, social participation, home integration, and the ability to go back to school [10, 11].

### Integrated care in traumatic brain injury

In 2016, the World Health Organization (WHO) released the “Framework on Integrated People-Centered Health Services,” which promotes a vision whereby “all people have equal access to quality health services that are co-produced in a way that meets their life course needs coordinated across the continuum of care” [12]. The WHO defines integrated care as “an approach to strengthen people-centered health systems through the promotion of the comprehensive delivery of quality services across the life-course, designed according to the multidimensional needs of the population and the individual and delivered by a coordinated multidisciplinary team of providers working across settings and levels of care” [12]. In this definition, the WHO advances concepts such as people-centered and multidimensional teams working across care settings. However, this definition fails to grapple with the unequal distribution of wealth, resources, and power that skews towards white Europeans (i.e., regardless of whether they are within or outside of Europe) and away from displaced Indigenous peoples globally. Health is unequally distributed and operates on the basis of power relations [2, 3, 13, 14]. For instance, Linton and Kim [15] performed a secondary data analysis finding that Black and Indigenous populations are disproportionately affected by intentional TBI irrespective of age and gender.

Integrated care can also be described as integrated care pathways, coordinated care pathways, care maps, connected care pathways, or predicted recovery pathways [16]. These synonymous processes describe the pertinent steps required in the care for particular clinical populations and chronicle the patient’s predicted clinical course [16]. For the purposes of this inquiry, integrated care pathways refers to, “the tasks to be carried out together with the timing and sequence of these tasks and the discipline for a specific clinical condition” [17]. This definition of integrated care leaves room for a critical examination of the care journey.

### Traumatic brain injury, racism, and Blackness

Racism stems from a historically constructed position of whiteness and embodies a social location of power that marks the social category of white as the locus of social privilege [18]. Racism is a social determinant of health because it contributes substantially to social inequalities such as poverty, unequal job access, and subsequent living conditions [19]. Racism further reconfigures health behaviors, access to health care, and interactions with various health-care professionals [19–23]. Rather than focusing on the social conditions and processes of racism that produce adverse health outcomes, health researchers and clinicians often credit racial disparities in health to biological differences based on race; this is on the basis of human constructed racial categories and unscientific claims for genetic variation and phenotypic distinctions [24–26]. Whiteness has been constructed to be natural and normal and that everything different to it is depicted as the other as well as lesser [18]. However, there is no biological basis for whiteness or blackness, and no particular genetic characteristics that are shared by only Black people and not white individuals, or vice versa [27].

Race is a socially created category of identity with no biological basis [28]. The concept of race (i.e., whiteness and blackness) was created by people with white skin to justify and legitimize subordination and superiority over those considered to be non-white, most notably in the forms of colonization and slavery [29]. Table 1 further defines these key concepts. As stated by Ta-Nehisi Coates [30], “…race is the child of racism, not the father.” Racialization is a fundamental organizing factor of society that consequently organizes everyday aspects of life including the economic, political, and social arenas [18, 28].

**Table 1:** Defining key concepts

Concept	Definition
Race	• Races are socially constructed categories that are not biologically defined but were designed and continue to produce advantages for some and disadvantages for others [28]. • Race is an organizing doctrine that dictates the social relationships that people have with one another [31]. • Race reshapes a person’s identity at an individual (micro) level in relation to white individuals and their social and geographical positioning which forms the rest of interpersonal life at a larger (macro) level scale [31].
Racism	• Racism, also termed racial ideology, provides the basis for disparities among various races on economic, social, and political bases [28]. • Racism depends on racialization and provides the instructions and justification on how individuals operate within systems and institutions according to racial categories [31]. • Racism towards people of colour eventually becomes normal, “common sense” [31], and serves a purpose. How racism operates in the world mirrors the ways in which systems and institutions function [31].
Racialization	• Racialized social systems create hierarchical interpersonal relationships between races such as white individuals and people of color [28]. • Racialization, which includes racialized social systems and social organizations, is a process that governs the assignment of differential benefits on an economic, social, and political basis according to socially defined categories of identity such as white and Black [28]. • Racialized social systems remain constant through ‘colour blind’ ideologies that ignore racism and instead open up discussions about disparities on other bases, such as classism [32].

Blackness, and anti-Black racism, are an important part of this picture. Richard Iton [33] describes Blackness as lacking a conclusive definition, noting: “rather, it is the interpretations, visions, and practices given to it by subaltern populations and a ‘category of underdeveloped possibilities.’” Put differently, Blackness is a political, historical, contemporary, and culturally constructed phenomenon that holds meaning and tangible consequences dependent on the context (i.e., date, location, form, and function of social and economic institutions). Blackness is an umbrella term for articulating all the nuances of what it means to retain a whole, human, and valuable identity in the midst of racialization and ongoing experiences of racism.

Current understandings of mechanisms of injury and community integration in integrated care fail to question the existence of racialized social systems and how anti-Blackness operates in a particular geographical context to consciously produce and perpetuate adverse and differential effects. It is difficult to understand how and what community integration means for Black populations without broader discussions of how whiteness, geography, racism, and racialization have shaped the contexts that Black populations inhabit. Community integration is defined by Sander et al. [34] as social participation, home integration, and productivity and is depicted as the goal of rehabilitation post-TBI. When discussing Blackness and community integration in TBI, the standpoint of whiteness and its embodiment come into question as whiteness continually produces values in which the rest of the world operates [18]. It is also important to acknowledge that the concept of productivity has particular contradictions for Black people given geography and the history of forced free labour that fueled the modern market economy [35, 36], and ongoing trauma of the transatlantic enslavement trade [37]. Furthermore, health is often presented as an individualistic phenomenon between the clinician and patient. This individualistic approach to health obscures structural forces, like racism that produce population effects that occur within day to day life, and accumulate over the life course. Therefore, individualistic definitions of health do not account for the primary conditions that produce poor health, poor community integration, and “unproductivity” for Black populations [38]. Racism and racialization have material effects on health [39]. Moreover, the capacity to comprehend the reality of Black people is significantly limited if the framework of reference is based on the inference of non-Black reality [40].

### Critical transdisciplinarity as a methodological framework and analytical tool

The practice of transdisciplinarity offers a holistic and coherent approach to research, which Pohl [41] argues should be considered for producing knowledge and practices that advocate for a “common good.” However, transdisciplinarity requires critical inquiry beyond the researcher and research process [41]. To this end, critical transdisciplinarity (CTD) [42] is a transformational methodological framework and analytical tool offering a paradigmatic shift toward a realist onto-epistemological worldview that is guided by four bodies of theories including the theory of political economy, critical political ecology, critical race theory, and intersectionality. In all cases, power is at the center of focus. Furthermore, Patricia Hill Collins’ uses the term “matrix of domination,” which highlights the intricacies of how people experience privilege and oppression, and how they silently operate within society to uplift some while simultaneously repressing others [43, 44]. What does occupational justice mean for rehabilitation and integrated care in the absence of consideration of the detrimental effects of racial capitalism and social and economic deprivation? Table 2 provides an overview of the theories underpinning a critical transdisciplinary approach. CTD elevates the focus of integrated care from person-centered care to a social justice, health equity lens that interrogates the ways in which intersecting processes operate to produce unequal outcomes. In this review, CTD is intended to serve as an impetus for using a social justice lens to question and mitigate adverse outcomes for underserved populations, and Black people in particular.

**Table 2:** Overview of the theories underpinning a critical transdisciplinary approach

Theory	Description	Seminal texts	Sample questions raised by examining integrated care through this lens
Political economy	• Political economy centers power and the distribution of resources (i.e., access to money, participating in commerce at the individual and global level); it focuses on the interconnectedness between the state, economy, and the rest of society [14]. • The political nature of the economic system functions to exclude people on the basis of ability, race, gender, class, social groups, organizations, and countries creating a hierarchical social order that is contingent on its success [14]. • A political economy lens shines a spotlight on how economic systems, such as capitalism and communism, perform in reality to better understand structures of power and the experiences of individuals through the matrix of domination [3, 37, 45] .	• The Political Economy of Health: A Useful Theoretical Tool for Health Education Practice [14]. • The Persistent Power of “Race” in the Cultural and Political Economy of Racism [46]. • The Political Economy of the Disability Marketplace [47]. • Black Political Economy in the 21st Century: Exploring the Interface of Economics and Black Studies—Addressing the Challenge of Harold Cruse [48]. • The Disability Business: Rehabilitation in America [49].	• Why do some populations of people or geographic sites around the world have better health outcomes than others? • How do different health-care systems shape health inequities? • Why do some of the wealthiest countries have relatively poor health outcomes despite their power, status, and resources? • Who benefits from particular forms of integrated care and who is left out?
Critical political ecology	• Critical political ecology aims to comprehend ecological reality by merging political economy with the politics of the built environment and the geography of whiteness [50]. • Interrogates and exposes hidden politics of political ecology that transcend epistemological boundaries, highlighting the politics that operate to produce particular living conditions, built environment, and environmental change [50]. • Critical political ecology is contingent on a historical analysis to better understand and illuminate past hidden and current dynamics between individuals and their ecological environment [13, 51].	• The Political Ecology of Disease as One New Focus for Medical Geography [13]. • Third World Political Ecology [51]. • Critical Political Ecology: The Politics of Environmental Science [50].	• Why is the prevalence of TBI higher in particular geographical contexts? • Why is the prevalence of TBI higher in different populations in the same geographical context? • How does the geographical and historic context of the injury contribute to the disease experience and health outcomes?
Critical race theory	• Originated in the field of law as a reaction to the absences of race within critical legal studies. • Defined as the collective work of African American legal scholars advocating for the development of a body of theory that recollects and displays the role of racism in American law which could be applied to end all forms of racism and subordination [52]. • Interrogates and re-thinks how racism is viewed as a social determinant of health to fill gaps in current understandings by viewing it from the perspective of what influence peoples’ living conditions such as occupational attainment, housing, and access to health services [53]. • Foregrounds and challenges racism as a normalized process that largely goes unacknowledged and unaddressed [32].	• The Key Writings that Formed The Movement [54]. • Critical Race Theory: Past, present, and future [55]. • Critical Race Theory: An introduction [56].	• Why is there is a higher prevalence of violence and TBI in Black communities? • Why are Black individuals reported to score lower on community integration? • How is race considered within the methodology of TBI research, and what is the impact of the conceptualization of racism on study outcomes? • What kind of information is collected about race and how is it associated with integrated care? • How is race-related information linked to context for understanding why certain racialized groups have differential outcomes in community integration?
Intersectionality	• Concerned with how multiple systems of oppression work together to produce new, complex categories of suffering [57] known as interlocking oppression [58]. • Rejects the idea of singular identities, i.e., that race, gender, ability or class may be understood as separate, unconnected categories [43, 57, 59].	• Demarginalizing the Intersection of Race and Sex: A Black Feminist Critique of Antidiscrimination Doctrine, Feminist Theory and Antiracist Politics [60]. • Mapping the Margins: Intersectionality, Identity Politics, and Violence Against Women of Color [57].	• What aspects social identity are collected in the research process, and how are they understood to be interconnected? • To what extent are multiple intersectional forms of identity and their processes used to interpret the findings? • How might outcomes be different had there have been consideration for the multiple intersecting identities of the participants? • How do differential health outcomes reflect the ways in which individuals with particular social identities experience integrated care?

### Rationale statement

A one-size-fits-all approach that attempts to see the struggle of all minorities as a single and isolated operation to address health inequalities is harmful. For this reason, it is important to classify anti-Black racism as separate from the experiences of other racialized groups. The extent, nature, and range of integrated care pathways for persons with TBI have never been comprehensively reviewed with a health equity lens and, in particular, for the purposes of illuminating how Black people are presented in the literature.

### Objectives of this review

It is important that health-care professionals, rehabilitation clinicians and scientists, and policy makers apply a health equity lens to illuminate how health-care systems operate and are structured, including examing the purpose that care pathways and integrated care serves, and for whom. To help close the equity gap, this paper outlines the first protocol of a CTD scoping review that will interrogate and map the field of integrated care and Black populations experiencing a TBI. This review seeks to map the scope of the field in the literature that explicitly examines integrating care with respect to racialization, racism, and Blackness. This CTD scoping review will use a holistic and critical approach to explore the state of the literature regarding the sequencing of care and what happens to Black people as they enter the health-care system, rehabilitation care, and health-related research. This review of the literature will identify knowledge gaps and areas of future exploration, and will contribute to the long-overdue review of the breadth and nature of the literature on integrated care for Black persons experiencing TBI.

## Methods

### Protocol design

When appraising the various systematic approaches to reviewing the literature, the scoping review methodology was selected as the best method to map the literature on integrated care for Black persons with TBI. Scoping reviews offer an opportunity for critical examination. Critical transdisciplinarity/CTD operates from a realist onto-epistemological worldview. The ontological tenants of the realist paradigm articulate that “the structures creating the world cannot be directly observed” [61]. Realism displays that illustrating and understanding the mechanisms of particular processes such as racialization require knowledge of historical facts and occurrences and interpretation through the application of appropriate bodies of theories [61]. CTD is a realist perspective that views society and structural forces through the following four analytical lens, see above for Table 2. CTD embodies the requirements of a realist worldview as it functions on the basis of four bodies of theories that together think through what types of transformational practices are needed while taking into account what is already known. As a realist perspective, CTD questions the assumptions at play and the root causes, so that meaningful long-term investments can be made that transform the conditions, processes, and outcomes that are favorable for Black populations that experience social and economic disenfranchisement and displacement.

The application of CTD is both a rigorous methodology and theory. Brink [62] defines rigor as ensuring reliability and validity as two fundamental ingredients to the quality of research. CTD defines rigor similarly. It is important to understand that current standards in scoping and systematic reviews often lack transparency in detailing and describing the steps and decisions made to include or exclude literature that is incorporated in the review. Inclusion and exclusion criteria are often meant to serve as a guide when screening the literature and are not to be used to make definitive conclusions. It is the theoretical development and understanding of the relevant constructs that allow individuals to make the decision of what literature is included or excluded to answer the question; the criteria are meant to be used as a guide. As such, CTD challenges contemporary conventions and fills gaps to go beyond the listing of inclusion and exclusion criteria. When engaging in article screening, the terms and their comprehensive development, along with how the terms are contextualized are not often fully transparent in scoping studies. CTD seeks to contextualize and flesh out terms. During the scoping study, a documented record will be kept of the screened articles and decisions made to include or exclude the pertinent literature at all levels of screenings. This documentation will ensure that a high degree of rigor is met and will ultimately improve quality.

**Fig. 1:**
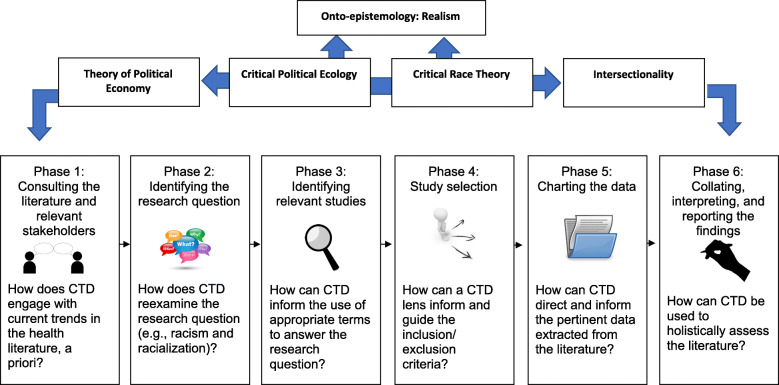
The critical transdisciplinary (CTD) scoping review process

This CTD scoping review protocol was developed under the methodological guidance of Akrsey and O’Malley [63], later refined by Levac, Colquhoun, and O’Brien [64], and further guided by the Joanna Briggs Institute [65]. The CTD scoping review process is displayed in Fig. 1. The 2018 Preferred Reporting Items for Systematic reviews and Meta-Analyses extension for Scoping Reviews (PRISMA-ScR) checklist [66] was also applied when developing the protocol for this CTD review. Please see Additional file 1 for the PRISMA-Protocol (PRISMA-P) checklist.

### Stage 1: consulting the literature and relevant stakeholders

The current standard for scoping reviews [64] often include this step as being optional but also as the last phase in conducting a scoping study. For instance, Levac et al. [64] articulate that this last stage of conducting a scoping review, namely consultation with relevant stakeholders, gives way to the opportunity to involve the input of other individuals who will provide insight beyond what is reported in the literature. CTD marks this phase as the first stage in conducting a scoping study. As such, prior to establishing the need for a scoping study, CTD comprehensively engages with current trends in the health literature in recognizing public health concerns as indicated by the prevalence and incidence of disease, differential outcomes, and disproportionate health disparities. Various community partners working with survivors of violence experiencing TBI expressed interested on knowing more about integrated care for persons with TBI. These conversations and discussions influenced and led to the application of the health equity lens of CTD for this review. However, this study will not include an additional consultation following analysis.

### Stage 2: identifying the research question

In accordance with CTD and to best meet the objectives of this scoping review, the following primary research question is asked: what is the extent, nature, and range of the literature on integrated care for Black people experiencing TBI? The research sub-questions are:

1. How are Blackness, race, and racism conceptualized in the literature on integrated care for Black people experiencing TBI?
2. How are the concepts of sex and gender positioned in relation to Black people generally and integrated care for Black people experiencing TBI specifically within the included texts?
3. How do Black people experiencing a TBI come to access integrated care pathways?
4. How do integrated care pathways for Black persons experiencing a TBI take into account the mechanism of injury (i.e., interpersonal violence) and the implications for occupation?

### Stage 3: identifying relevant studies

#### Sources of information

To identify relevant studies for this CTD scoping review, the following electronic databases of the published peer-reviewed literature will be systematically searched including MEDLINE (In-Process and Other Non-Indexed Citations), EMBASE, Cochrane Central Register of Controlled Trials, Cumulative Index to Nursing and Allied Health Literature (CINAHL), PsycINFO, and Sociological Abstracts. This study will not include books or grey literature.

#### Search strategy

The search strategy for this review was developed through an iterative process in collaboration with an Information Specialist. The search strategy included the application of text words and subject headings (e.g., MeSH, Emtree) in congruence with the following concepts: TBI, integrated care, and Black populations. The terms used for this review were informed based on other reviews on integrated care [17, 67–74], the WHO’s “Framework on Integrated People-Centered Health Services” [12], and other reviews on racism and racialization [19, 75, 76].

The complete and final search strategy in MEDLINE can be found in Additional file 2. The reference lists of the included articles will be hand searched to identify any key sources of information that may have been missed by the search strategy. Limits will also be placed on the search strategy to restrict studies to published literature from 1980 and onwards as this is when the term integrated care first appeared in the health literacy [77]. The searches will also be limited to English language publications. This review will be undertaken without any restrictions to geographical location as the intention is to map the breadth of the literature globally.

### Stage 4: study selection

#### Eligibility criteria

Given the above operationalization of integrated care pathways, the following inclusion and exclusion criteria will be used:

##### Inclusion criteria

1. All texts must include a description of an “integrated care pathway” or integrated care, which may be a plan, model, protocol, or intervention that maps onto the pre-identified definition, integrated care pathways refers to, “the tasks to be carried out together with the timing and sequence of these tasks and the discipline for a specific clinical condition” [37] across all service delivery settings (e.g., community-based care, community-based agencies, community-based organizations, acute/subacute care institutions, and rehabilitation hospitals).
2. The integrated care pathway must be considered in the context of persons with a primary diagnosis of TBI of any level of severity.
3. The persons with TBI must include Black persons in the sample or as part of the text for non-empirical articles and/or any of the words race, racism, racialization, and Blackness.
4. The text must be published in English.
5. The text must be peer-reviewed literature.

##### Exclusion criteria

1. Other neurological conditions that are not indicated as being a sequelae of TBI

The review process in this scoping review will consist of two levels of screening: (1) a title and abstract review and (2) full-text review. Prior to commencing the first level of screening, two independent reviewers will screen a sample of 20 abstracts against the criteria to compare and discuss any discrepancies, to increase consistency. The two reviewers will independently review the first level of screening against the inclusion and exclusion criteria. All articles that meet the criteria above will be included for the second level of screening which will consist of full-text review of the selected articles against the predetermined inclusion criteria. Where differences or any discrepancies arise after discussion between the two reviewers, a third reviewer will be consulted to reach consensus.

**Fig. 2:**
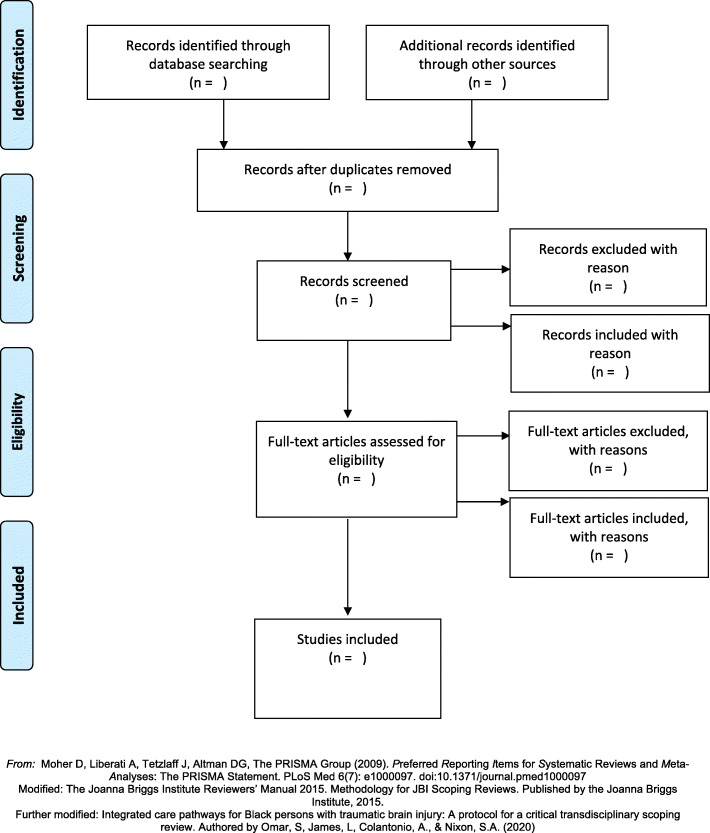
PRISMA flow diagram for the critical transdisciplinary (CTD) scoping review process

Please see Fig. 2 for a modified [65, 78] PRISMA flow diagram of the scoping review article retrieval process for retrieving articles.

### Stage 5: charting the data

After consensus is reached on which articles will be included in the review, the relevant information will be abstracted. To assist with data charting, a form has been designed a priori for this CTD scoping review and will be used to abstract data. The goal is to extract information that will help address the objectives and research questions for this review which have been highlighted in the protocol. Yet, current conventions on conducting scoping reviews often lack detail in their data charting forms and mainly abstract summary characteristics such as the author information, variables, and central findings. CTD is a comprehensive, holistic, and transformative tool that allows individuals to rigorously engage with the literature in a more detailed manner to improve the quality of work. As such, in creating the data charting form, the various elements of CTD including critical political ecology, political economy, critical race theory, and intersectionality were used to comprehensively inform the information required to answer the research questions. Please see Table 3 for the complete data charting form. Any additional information or comments from the reviewers will be documented in the form and will be used to add any themes or items that may not be captured in the current document. Similarly, any discrepancies in charting by the two independent reviewers will be discussed and consulted with a third reviewer, if appropriate.

**Table 3:** Data charting form for CTD scoping review objectives and research question(s)

Category	Variable
Summary characteristics	• Who are the author(s) and what are the years of publication? • What are the titles of the studies? • What is the study methodology (i.e., quantitative, qualitative and/or mixed methods including ethnography, phenomenology, review, or commentary)? • What are the authors’ stated objective(s)? • What is the geographical location of the study • What are the funding sources of the study? • What is the type of health system (federal, state, and provincial)?
Characteristics of the sample (for empirical texts), or who the authors are writing about (for non-empirical texts)	• Who are the participants (age, sex/gender, and race)? • What are the inclusion/exclusion criteria of the study population? • How do the authors define race? • How do the authors define Black? • How do the authors define and/or discuss sex and gender in relation to the Black participants? • What is the socioeconomic status of the participants? What is the mechanism of injury? • What is the severity of injury (mild, moderate, and severe)? • What is the time since the injury? • Where are the participants coming from and/or going to (i.e., hospital-based care, community-based care, return to home)?
Elements of the integrated care pathway (characteristics and persons involved)	• How is integrated care pathway described (i.e., integrated services, integrated collaborative practice, integrated organizations level, or systems level integration)? • What is the trajectory followed by Black people to obtain care (i.e., what are the pathways to care for Black people)? • What are the setting(s) for the integrated care pathway (inpatient, outpatient, community-based care, primary care)? • Which designated (i.e., clinicians) and non-designated (i.e., chaplains, caregivers, family, and/or peer supports) providers are part of this integrated care pathway (racial/ethnic characteristics) and what are their roles? • How does the integrated care pathway consider the mechanism of injury (i.e., interpersonal violence, accident, self-inflicted)? • How does the integrated care pathway consider occupation?
What is the integrated care pathway addressing?	• How does the integrated care pathway address the person (i.e., cognitive, affective, and physical)? • How does the integrated care pathway consider spirituality? • How does the integrated care pathway consider the environment (i.e., cultural, institutional, physical, and social spaces)?
Outcomes, barriers, and facilitators to the integrated care pathway	• What do the authors report as the main findings related to the integrated care pathway? • What are any reported barriers and/or facilitators to the integrated care pathway, and for whom (i.e., are they based on reported perceptions such as attitudes, beliefs, values, and/or knowledge)? • What claims do the authors make about Black people? • How are Black people included in the analysis? • How does anti-Blackness show up in the studies? • Is sex and/or gender included in the analysis about Black people? • What conclusions are reached about the mechanism of injury for Black people, and what are the implications for occupation?

### Stage 6: collating, interpreting, and reporting the findings

Where possible, the extracted data will be reported using descriptive statistics. For example, frequency tables may be used to provide a comparative analysis between study locations, and to better understand the representation of Black populations reported in integrated care pathways for persons with TBI. Scoping reviews often include an additional analysis piece that provides a qualitative analysis that reports about themes and patterns [63]. Rather than summarizing and reporting the findings in regards to patterns and themes, CTD uses four key bodies of literature for assessing the literature and interpreting the findings: (1) political economy, (2) critical political ecology, (3) critical race theory, and (4) intersectionality to contextualize. CTD also engages with the literature in a manner that recognizes, acknowledges, and addresses the contemporary and historical occurrences that contribute to ongoing health disparities affecting Black populations.

## Discussion

Integrated care has become an attractive “solution” to health-service delivery that has been explored in many chronic conditions. A current challenge of integrated care/integrated care pathway is the lack of consideration for Black lives. This makes it increasingly challenging to understanding what integrated care means for Black people experiencing TBI. In keeping with the current vision of equity and health for all [79], this protocol outlines the first CTD scoping review that will critically appraise the peer-reviewed literature on what is known about integrated care pathways and Black people who experience TBI. The application of CTD will aid in mapping the key elements, gaps, and outcomes of integrated care pathways. Furthermore, applying CTD will also examine where, how, and if Black people are considered, as well as forces known to negatively impact on health such as racism and racialization. In producing the first CTD scoping review, this paper contributes to the advancement of research on integrated care pathways for Black persons experiencing TBI. Future research may consider the implications of a CTD lens in other racialized populations that experience TBI or other neurological conditions.

## Strengths and limitations

This review will only examine literature published in English. Unfortunately, the vast majority of reviews exclude literature due to language barriers [80] and this limits the potential to learn from and have a better understanding of the field from many places around the world. Future reviews may benefit from including literature published in languages other than English to be more inclusive of other important contributors and geographical contexts that may have been excluded in this study. As a possible solution, this may involve the use of high-quality translation applications and collaborative-multilingual local research teams. Lastly, this review does not examine evidence from the grey literature. Grey literature involves information that extends beyond the scope of traditional mechanisms of publishing and communicating information. This inquiry intentionally sought to scope literature published in peer-reviewed sources in order to understand traditional perspectives regarding what is known about the integrated care pathways and Black people who experience TBI. Future research may choose to expand the research question to include grey literature and/or be inclusive of other languages.

A known limitation to scoping reviews is the lack of critical appraisal [63]. The application of a critical health equity, social justice lens helps overcome the lack of critical appraisal that is known to limit scoping reviews. Moreover, this review also considers the mechanism of injury, the presence and absence of racism, racialization, Blackness, intersections with sex and gender, and the extent to which they are reflected in the peer-reviewed, English language literature published between 1980 and 2019 in regards to integrated care pathways for people who have experienced TBI. The findings from this review may contribute to the development of new initiatives and policies for holistic, equitable care pathways by applying a critical-health equity lens to better support Black people living with a TBI.

## Supplementary information

**Additional file 1.** PRISMA-P Checklist.

**Additional file 2.** Medline search strategy.

## Data Availability

Further information related to this review can be provided upon reasonable request. Interested readers should contact the corresponding author.

## Abbreviations

CDC: Center for disease control and prevention
CTD: Critical transdisciplinary or critical transdisciplinarity
PRISMA-P: Preferred reporting items for systematic review and meta-analysis protocols
PRISMA-ScR: Preferred reporting items for systematic reviews and meta-analysis extension for scoping reviews
TBI: Traumatic brain injury
WHO: World health organization
