# Supplementary material for: Integrated care pathways for Black persons with traumatic brain injury: a protocol for a critical transdisciplinary scoping review
Source: Syst Rev. 2020 Jun 1;9:124. doi: 10.1186/s13643-020-01323-8 (PMC7265630; doi:10.1186/s13643-020-01323-8)
Supplement: Supplementary file 2 — Additional file 2. Medline search strategy. [file 13643_2020_1323_MOESM2_ESM.docx]

**Database: Ovid MEDLINE: Epub Ahead of Print, In-Process & Other Non-Indexed Citations, Ovid MEDLINE® Daily and Ovid MEDLINE® <1946-September 16^th^, 2019>**

**Search Strategy:**

--------------------------------------------------------------------------------

| Medline Search Strategy |
| --- |
| 1 exp Head Injuries, Closed/ (10962) |
| 2 exp Brain Injuries/ (65708) |
| 3 exp Brain Injuries, Traumatic/ (12172) |
| 4 exp Brain Concussion/ (8143) |
| 5 Craniocerebral Trauma/ (21782) |
| 6 tbi*2.tw,kf. (25224) |
| 7 mtbi*2.tw,kf. (2604) |
| 8 wrTBI*2.tw,kf. (13) |
| 9 concuss*.tw,kf. (8738) |
| 10 postconcuss*.tw,kf. (1297) |
| 11 ((brain or head* or cerebr* or crani* or skull* or intracran*) adj2 (injur* or trauma* or damag* or wound* or fracture* or contusion*)).tw,kf. (136377) |
| 12 ((brain* or cerebr* or intracerebr* or crani* or intracran* or head* or subdural* or epidural* or extradural*) adj (haematoma* or hematoma* or hemorrhag* or haemorrhag*)).tw,kf. (47624) |
| 13 or/1-12 (208936) |
| 14 exp "Delivery of Health Care, Integrated"/ (12123) |
| 15 ((integrat* or coordinat* or co-ordinat*) and (care or health care or healthcare or service? or treatment? or therap* or program* or path? or pathway?)).tw,kf. (293443) |
| 16 (integrat* adj3 (care or health care or healthcare or service? or treatment? or therap* or program* or path? or pathway?)).tw,kf. (40514) |
| 17 (connect* adj3 (care or health care or healthcare or service? or treatment? or therap* or program* or path? or pathway?)).tw,kf. (8297) |
| 18 (coordinat* adj3 (care or health care or healthcare or service? or treatment? or therap* or program* or path? or pathway?)).tw,kf. (17271) |
| 19 ("Life care plan?" or "Life care planning").tw,kf. (193) |
| 20 Patient Care Team/ (63297) |
| 21 case management/ (9885) |
| 22 patient care management/ (3759) |
| 23 comprehensive health care/ (6508) |
| 24 patient-centered care/ (18055) |
| 25 ((interdisciplin* or inter-disciplin* or interprofession* or inter-profession* or multidisciplin* or multi-disciplin* or multiprofession* or multi-profession* or transdisciplin*) adj3 (team or care or health care or healthcare or service? or treatment? or therap* or program*)).tw,kf. (43422) |
| 26 patient care planning/ (37907) |
| 27 (help-seeking adj3 duration).tw,kf. (8) |
| 28 (help seeking adj3 experienc*).tw,kf. (184) |
| 29 (help-seeking adj4 model).tw,kf. (79) |
| 30 (treatment adj4 delay*).tw,kf. (22792) |
| 31 (system adj3 delay*).tw,kf. (1702) |
| 32 (systemic adj3 delay*).tw,kf. (590) |
| 33 (referral adj3 delay*).tw,kf. (904) |
| 34 (contact* adj3 professional).tw,kf. (585) |
| 35 (care adj3 delay).tw,kf. (1216) |
| 36 journey of care.tw,kf. (35) |
| 37 care contact*.tw,kf. (466) |
| 38 point* of entry.tw,kf. (1051) |
| 39 (entry adj2 service*).tw,kf. (118) |
| 40 or/14-39 (483232) |
| 41 somali american.tw,kf. (7) |
| 42 somali canadian.tw,kf. (2) |
| 43 carribean american.tw,kf. (0) |
| 44 afro-cuban.tw,kf. (3) |
| 45 Afro-Brazil*.tw,kf. (139) |
| 46 Afro-Haiti*.tw,kf. (2) |
| 47 Afro-Latin*.tw,kf. (11) |
| 48 Afro-Argentin*.tw,kf. (1) |
| 49 Afro-Bolivi*.tw,kf. (3) |
| 50 Afro-Chil*.tw,kf. (0) |
| 51 Afro-Colombia*.tw,kf. (51) |
| 52 Afro-Ecuador*.tw,kf. (17) |
| 53 Afro-Peruvi*.tw,kf. (0) |
| 54 Afro-Uruguay*.tw,kf. (2) |
| 55 Afro-Venezuel*.tw,kf. (1) |
| 56 Afro-Beliz*.tw,kf. (1) |
| 57 Afro-Costa Rica*.tw,kf. (1) |
| 58 Afro-Salvador*.tw,kf. (0) |
| 59 Afro-Guatemal*.tw,kf. (0) |
| 60 Afro-Hondur*.tw,kf. (0) |
| 61 Afro-Nicaragu*.tw,kf. (0) |
| 62 Afro-Panaman*.tw,kf. (4) |
| 63 Afro-Dominic*.tw,kf. (0) |
| 64 Afro-Puerto Rica*.tw,kf. (0) |
| 65 Afro-Mexica*.tw,kf. (0) |
| 66 Black-Hispanic*.tw,kf. (906) |
| 67 exp African Continental Ancestry Group/ (83295) |
| 68 West Indian.tw,kf. (820) |
| 69 Afro Caribbe*.tw,kf. (1242) |
| 70 Afro America*.tw,kf. (318) |
| 71 African America*.tw,kf. (51582) |
| 72 African Caribbe*.tw,kf. (657) |
| 73 Black.tw,kf. (117904) |
| 74 Afro-Carribe*.tw,kf. (13) |
| 75 afro-america*.tw,kf. (318) |
| 76 Race.tw,kf. (99900) |
| 77 exp racism/ (1955) |
| 78 or/41-77 (270458) |
| 79 13 and 40 and 78 (40) |
| 80 limit 79 to yr="1980 - 2019" (39) |
| 81 limit 80 to english language (37) |
| 82 81 not (exp animals/ not exp humans/) (36) |
